# Numerical analysis on the improvement of sound insulation performance of plenum doors: effect of sound absorption and MPPs

**DOI:** 10.14324/111.444/ucloe.3598

**Published:** 2026-05-28

**Authors:** Kazusa Kodama, Kimihiro Sakagami

**Affiliations:** 1 Environmental Acoustics Laboratory, Graduate School of Engineering, Kobe University, Japan

**Keywords:** plenum structure, sound insulation performance, finite element method, sound transmission loss

## Abstract

In recent years, the importance of natural ventilation has been increasingly recognised and achieving a balance between natural ventilation and sound insulation has become a key concern. This study investigates the sound insulation performance of plenum doors – openings designed to achieve both natural ventilation and sound insulation – using the finite element method. In particular, the study examines cases where sound-absorbing materials and micro-perforated panels are inserted into the plenum interior. Previous studies have evaluated the sound insulation performance of plenum doors; however, investigations are limited to specific absorption locations and those involving the introduction of micro-perforated panels have not been conducted. Partial application of sound-absorbing materials and the insertion of micro-perforated panels make it possible to maintain visibility and daylighting. In this way, plenum doors can provide benefits not only in terms of sound insulation but also in visibility and daylight performance. Sound-absorbing materials were applied to the interior surfaces of the plenum, and their effects were especially significant in the mid-to-high-frequency range, with transmission loss increasing by approximately 4–9 dB around 1000 Hz. The degree of improvement was found to increase with the absorption area and coefficient, while differences due to placement location were relatively small, on the order of 0.5–3 dB. The insertion position of the micro-perforated panels was varied and analysed. Micro-perforated panels were shown to enhance sound transmission loss in specific frequency bands. However, securing a sufficient air back space is essential to achieve this effect. Moreover, the effectiveness of micro-perforated panels depends on installation conditions such as surface area and placement, indicating the need for further evaluation.

## Introduction

In recent years, the importance of natural ventilation has been increasingly emphasised mainly due to the impact of infectious diseases. Ventilation offers a wide range of benefits, including the removal of viruses and pollutants, as well as the regulation of humidity and indoor temperature. Recently, studies have also highlighted the significance and necessity of natural ventilation [1], and it is not uncommon to see situations where windows and doors are left open for ventilation purposes. Furthermore, natural ventilation is considered advantageous compared to mechanical ventilation from the perspective of energy policy, and it also contributes to the improvement of indoor air quality. However, natural ventilation reduces indoor airtightness and can deteriorate the acoustic environment due to poor sound insulation. Problems such as decreased speech intelligibility due to external noise intrusion and leakage of indoor conversations and sounds may arise. As ventilation and sound insulation are closely related, it is essential to achieve a balance between them.

Plenum structures, originally used as silencers in air-conditioning ducts, are expected to enable both natural ventilation and sound insulation when applied to openings. Openings with plenum structures that achieve both natural ventilation and sound insulation have been widely studied, particularly in applications to windows (hereafter called ‘plenum windows’) [2–6]. Originally used as a silencing mechanism in air-conditioning ducts, plenum structures reduce sound through cross-sectional changes at the entrance and exit, along with internal acoustic treatments. In plenum windows, this structure is adapted by using two glass panes, each with staggered openings at their ends [7]. As for the other type of a window with natural ventilation, a different type of device is also proposed [8, 9].

On the one hand, when the same concept is applied to doors (hereafter called ‘plenum doors’), the sound insulation performance under variations in structural parameters has been investigated by Sakagami et al. [10]. Compared with plenum windows, plenum doors have dimensional constraints in their geometric configuration. In particular, limitations in thickness are significant, requiring careful considerations on the size of the air cavity between panels. Furthermore, as doors are generally taller than windows, designing a practical structure is more challenging. On the other hand, unlike windows, ensuring transparency in doors is not essential. This allows for a variety of measures to improve sound insulation performance, such as the introduction of sound-absorbing materials and micro-perforated panels (MPPs). Consequently, a wide range of application possibilities can be explored, offering greater flexibility and practicality.

In Sakagami et al. [10], the effects of parameters such as opening width, door width, cavity depth and the presence of sound-absorbing materials on the sound transmission loss of plenum structures were investigated through numerical analyses using the finite element method (FEM). To approximate realistic conditions, a diffuse incidence model of a plenum door was constructed. The results showed that smaller opening widths and shallower cavity depths lead to better sound insulation performance. Furthermore, applying sound-absorbing materials to the inner surfaces of the door panels was found to enhance sound insulation.

In this study, to further evaluate the sound insulation performance of plenum doors, numerical analyses of sound transmission loss were conducted using FEM, based on the model presented in Sakagami et al. [10].

First, the sound insulation performance of plenum doors treated with sound-absorbing materials while maintaining transparency was evaluated. Maintaining the transparency of doors is advantageous for visibility and daylighting, thereby expanding their architectural applicability. Moreover, by applying sound-absorbing materials only to part of the panels, sound absorption material can be saved, which is expected to offer advantages in terms of manufacturing cost.

In addition, the insertion of MPPs into plenum doors was investigated, and effective insertion methods were examined. The use of MPPs is expected to enhance sound insulation performance in specific frequency ranges. As plenum doors tend to exhibit a dip in sound insulation performance around 300 Hz, the insertion of MPPs is anticipated to improve performance particularly in this frequency band.

## Common aspects of the analysis model

**Figure 1:**
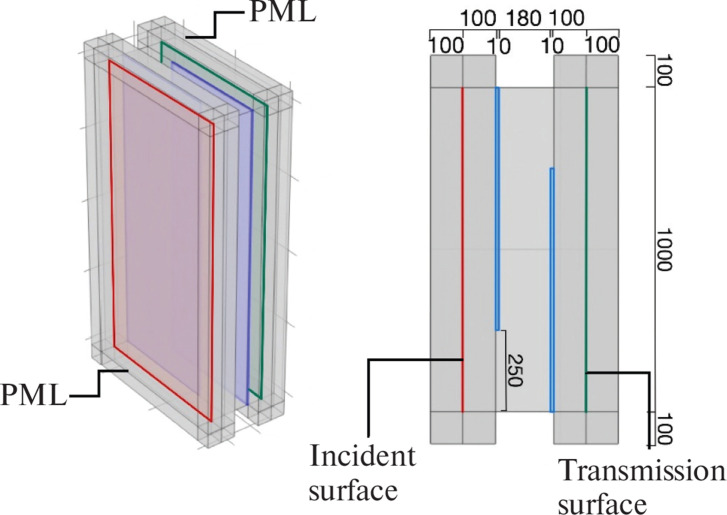
Analysis model of the plenum door.

In this study, sound insulation analysis of plenum doors was conducted using the FEM. The analysis utilised the Pressure Acoustics Module of COMSOL Multiphysics^®^ version 6.2 (COMSOL AB, Stockholm). As illustrated in Fig. 1, the plenum door consists of two panels (10 mm thick, shown in blue in Fig. 1) and an air cavity (2000 mm high, 1000 mm wide, 180 mm thick). These two panels simulate the front and back panels of the door, with staggered openings (250 mm wide) arranged alternately. Additionally, air layers (2000 mm high, 1000 mm wide, 100 mm thick) are placed on both the incident and transmission sides of the door.

All air regions are filled with air under standard conditions (1 atm pressure, 293.15 K temperature). At the red plane shown in Fig. 1, 300 plane waves with random wavenumbers are assumed to be incident in order to simulate diffuse sound incidence. These 300 plane waves have random propagation directions and phases. Convergence was confirmed at 300 waves after examining the cases with 100–500 waves.

The ratio of incident acoustic power (*Pi*) at the red plane to transmitted acoustic power (*Pr*) at the green plane is used to calculate the sound transmission loss (*TL* = 10 log|*Pi*/*Pr*|). To eliminate the influence of reflections from surroundings, perfectly matched layers (PMLs) were applied around the model. In the diffuse incidence model presented in Fusaro et al. [8], only the air layer on the transmission side was surrounded by PMLs. In this study, however, PMLs are also applied to the air layer on the incident side, thereby simulating reflection-free spaces both in front of and behind the door.

Unless otherwise specified, the analysis was conducted in the frequency range of 100–4000 Hz at 1/24-octave intervals, and the results were averaged every 1/6-octave. The mesh generation was performed using the meshing functions of COMSOL Multiphysics^®^. A free tetrahedral mesh was applied to the air layer inside the plenum door, while sweep meshes were used for the surrounding air region and the PML layer. The maximum and minimum element sizes were set to one-fifth (17 mm) and one-tenth (8.5 mm), respectively, of the wavelength corresponding to the upper analysis frequency of 4000 Hz, ensuring sufficient computational accuracy. The total number of mesh elements ranged from approximately 220,000 to 1,400,000, and the number of degrees of freedom ranged from approximately 820,000 to 3,790,000.

## Acoustic treatment on the interior surfaces on the periphery of the plenum

**Figure 2:**
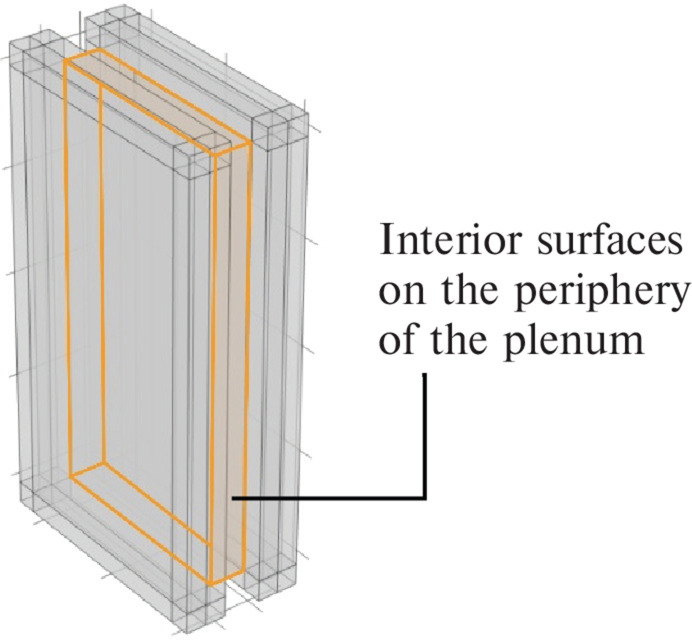
The interior surfaces on the periphery of the plenum door.

In this section, the sound transmission loss is examined when acoustic treatment is applied to the interior surfaces on the periphery of the plenum door. Impedance boundary conditions were assigned to the regions shown in Fig. 2, and the absorption coefficient was varied to investigate its effect on sound transmission loss.

### Absorption coefficient of the interior surfaces

To investigate the sound insulation performance when acoustic treatment is applied to the inner sides of plenum doors, impedance boundaries corresponding to normal incidence sound absorption coefficients of 0.1, 0.2, 0.3, 0.5 and 0.7 were applied to the interior surfaces of the model (the yellow surfaces on the periphery, as indicated in Fig. 2). As the purpose of this study is to investigate the change in sound transmission loss based on the absorption coefficient, the absorption coefficients assigned in the simulations are idealised values and are assumed to be constant with respect to frequency.

### Results and discussion

**Figure 3:**
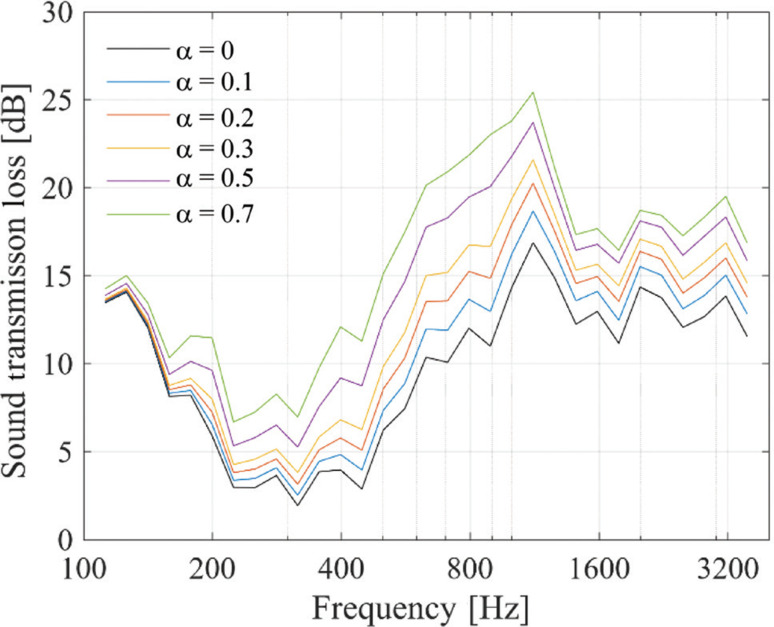
Sound transmission loss with sound absorption applied to the interior surfaces on the periphery of the plenum.

The results are shown in Fig. 3. When sound absorption was applied to the interior surfaces of the plenum door, the *TL* increased, particularly in the mid-frequency range. The *TL* increased with the sound absorption coefficient, showing that the level of increase was dependent on the coefficient. When focusing on the case with an absorption coefficient of 0.2, the increase in *TL* was approximately 4 dB at 1000 Hz. In the study by Sakagami et al. [10], where acoustic treatment was applied to the entire interior surfaces of both side panels (the blue panels in Fig. 1), the increase in *TL* at 1000 Hz with an absorption coefficient of 0.2 was reported to be approximately 12 dB, which is larger than the increase observed in the present study. The relatively small increase in *TL* obtained in this study is considered to be due to the smaller area of the sound-absorbing treatment, which was approximately 1.2 m^2^, compared with the area treated in Fusaro et al. [8], which was 3 m^2^.

## Acoustic treatment on the interior surfaces of the panels

This section investigates the sound transmission loss when acoustic treatment is applied to the interior surfaces of the plenum door panels (the blue panels in Fig. 1). To maintain the transparency of the door, absorption is applied to only half of the panels. The effect of varying the absorption location on sound transmission loss is examined.

### Conditions examined

The sound absorption coefficient is assigned to the interior surfaces of the panels on both the incident and transmission sides in the model for an investigation. To preserve the transparency of the door, the analysis assumes the acoustic treatment applied to only half of the panels. Impedance boundaries corresponding to a sound absorption coefficient of 0.5 are given, and their positions are varied to the top, bottom, left and right as viewed from the incident side. These cases are designated as Case A, Case B, Case C and Case D, respectively (Table 1).

**Table 1.** Absorption placement and conditions for each case

	Case A	Case B	Case C	Case D
Figure (acoustic treatment is applied to the blue areas)	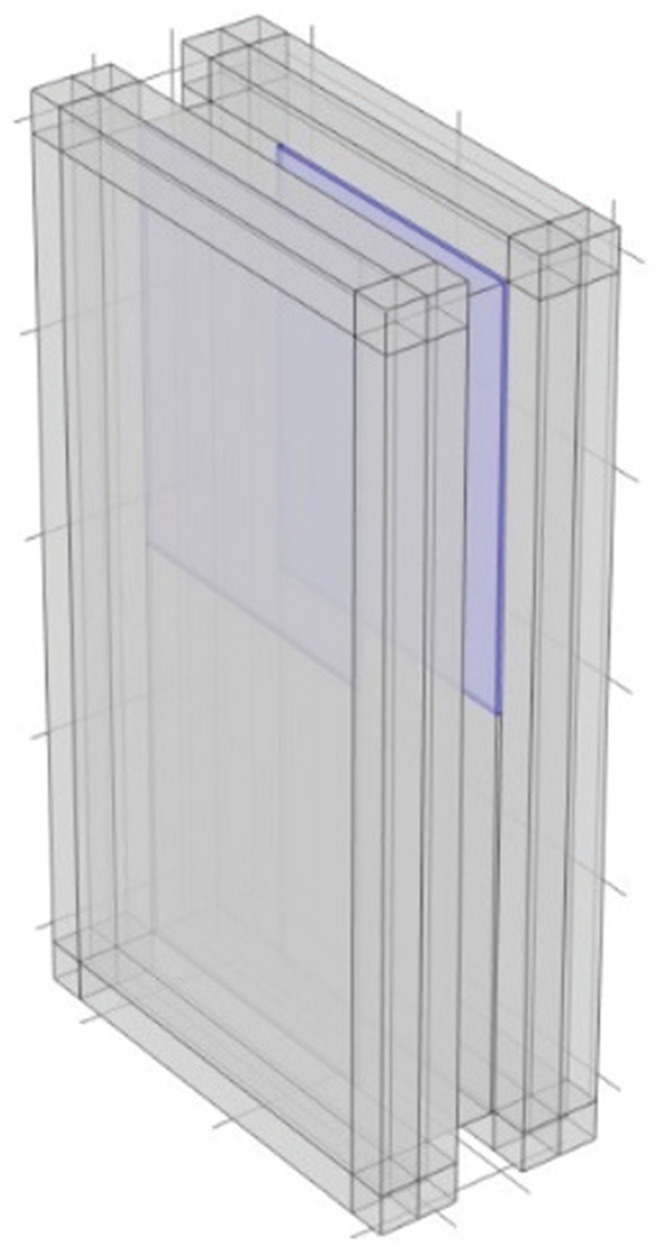	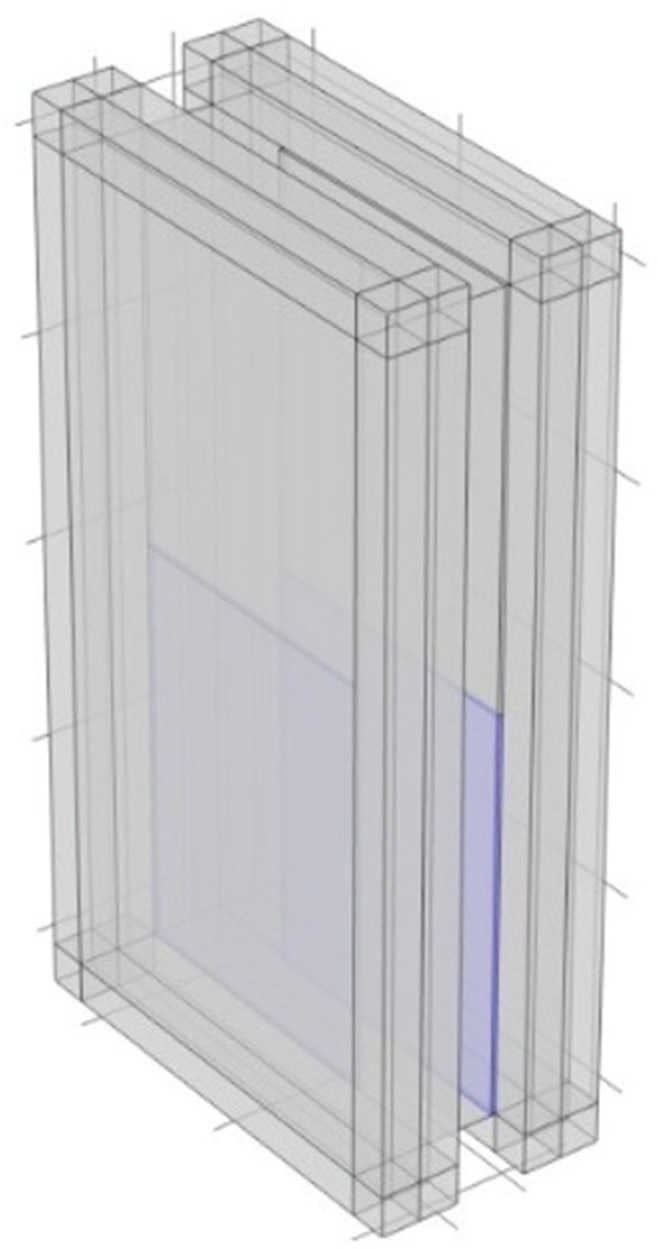	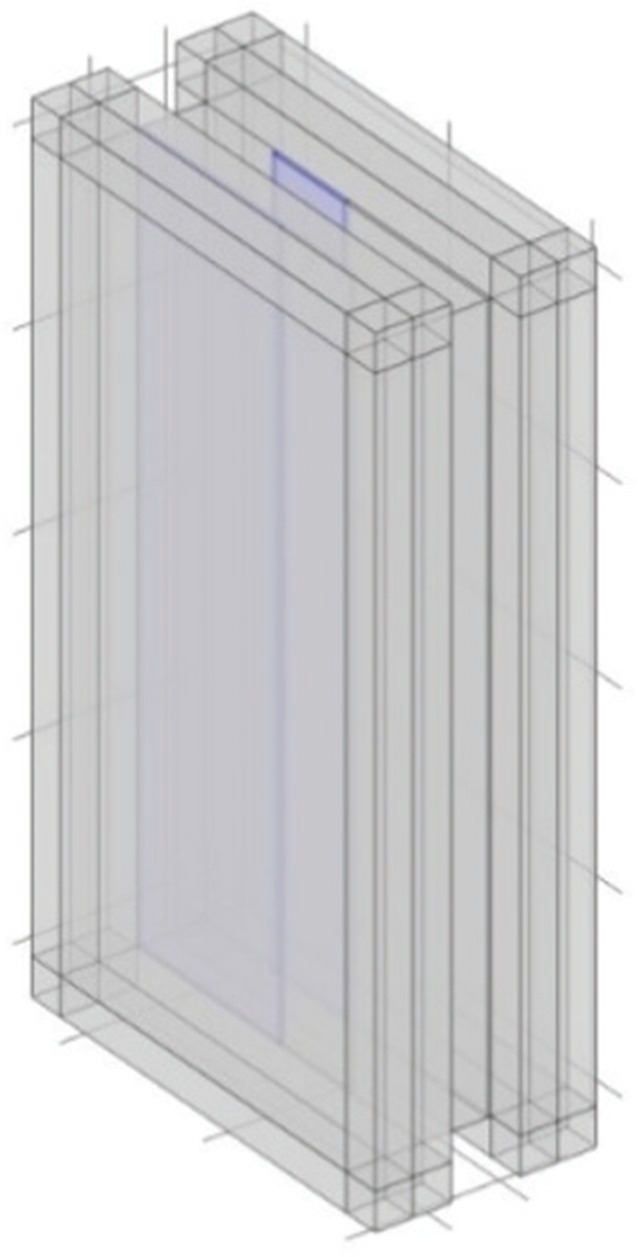	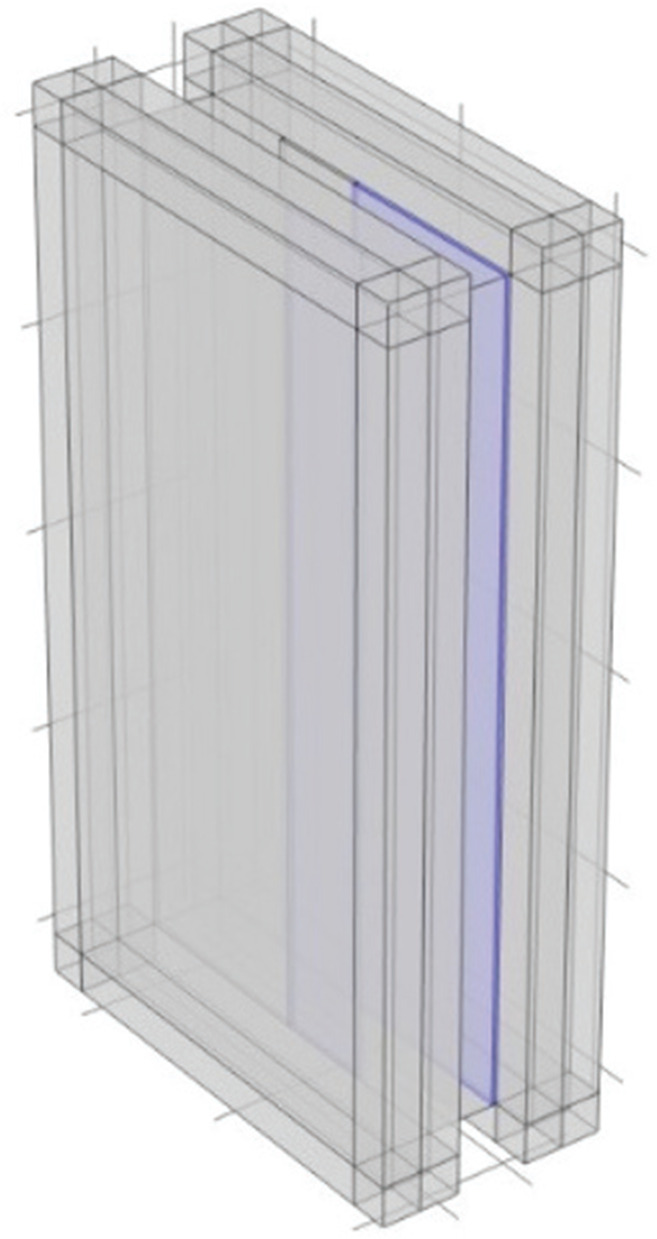
Placement of acoustic treatment	The top	The bottom	The left	The right
Absorption coefficient	0.5	0.5	0.5	0.5

### Results and discussion

**Figure 4:**
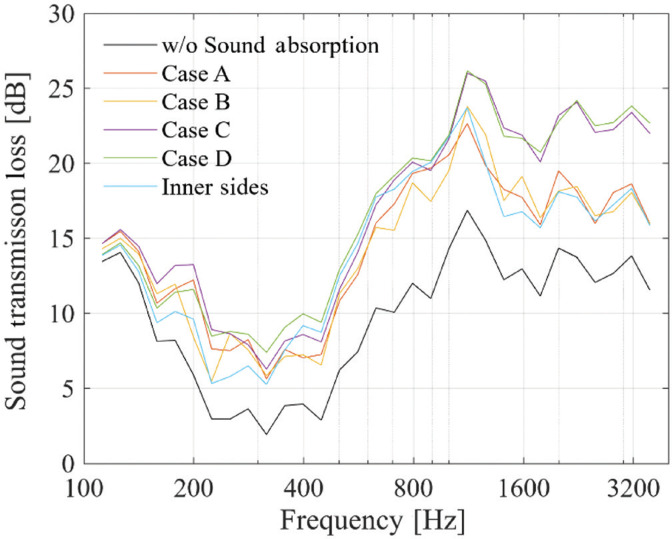
Sound transmission loss with sound absorption applied to the interior surfaces of the panels.

The results are shown in Fig. 4. When acoustic treatment is applied to the inner surfaces of the panels on both sides, an increase in *TL* of approximately 5 dB is observed, regardless of the position of the acoustic treatment. In terms of the amount of this increase, differences due to the treatment position are minimal – around 2–3 dB – in the low- and mid-frequency ranges. Additionally, when compared to the case in which acoustic treatment was made on the periphery of the plenum, no significant differences are noted. This is considered to be due to the small difference in the treated surface area.

In the high-frequency range, Cases C and D exhibit *TL* values approximately 5 dB higher than those of Cases A and B. This is attributed to the sound pressure distribution inside the plenum.

From these results, when prioritising door transparency, considering that the acoustic effects are of a similar level, acoustic treatment on the inner sides of the plenum is more advantageous.

## Installation of MPP to the surfaces on the periphery of the cavity

In this section, the surfaces on the periphery of the plenum door are modelled as MPPs, with air back spaces installed around it. The MPP is incorporated into the plenum door model to investigate whether its insertion can enhance sound insulation performance within specific frequency ranges.

### Examined conditions

**Figure 5:**
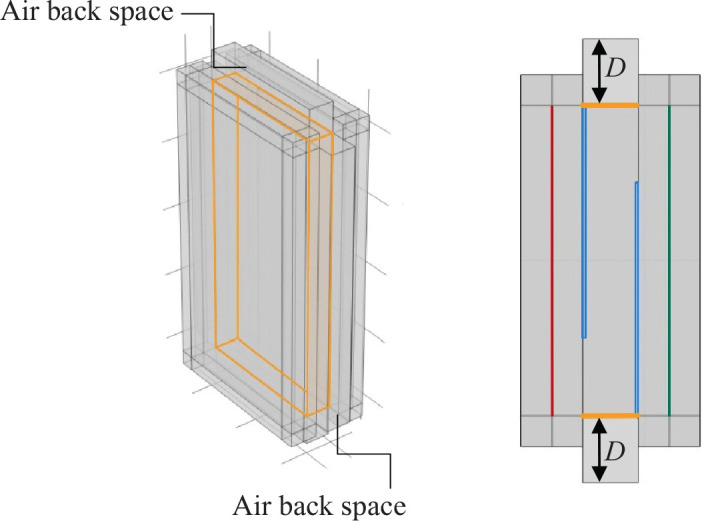
Model with MPP installed on the side portion (MPP1–4). *D* [mm] is the air back space thickness.

The surface on the periphery of the model described by the yellow surfaces in Fig. 5 were designated as MPPs. Surrounding spaces were introduced to create air back spaces, thereby integrating MPP into the plenum door (Fig. 5). The MPP was defined as an internal perforated plate boundary in the COMSOL model.

The depth of the air back space was varied – 300 mm, 200 mm, 100 mm and 50 mm – corresponding to MPP1 through MPP4, with each configuration assigned the parameters shown in Table 2. The parameters of the MPP listed in Table 2 were determined based on Maa’s theoretical model [11]. These values were specifically calculated to ensure that the peak absorption frequency aligns with the target frequency of approximately 300 Hz, where the plenum door’s sound insulation performance typically decreases. The analysis was conducted across the frequency range of 100–2000 Hz in 1/24-octave intervals, with the results averaged over 1/6-octave intervals.

**Table 2.** MPP parameter

MPP No.	Air back space *D* [mm]	Hole diameter *d* [mm]	Panel thickness *t* [mm]	Porosity *p* [%]
MPP 1	300	0.3	0.3	0.45
MPP 2	200	0.4	0.4	0.35
MPP 3	100	0.6	0.6	0.25
MPP 4	50	0.8	0.8	0.15

## Results and discussion

**Figure 6:**
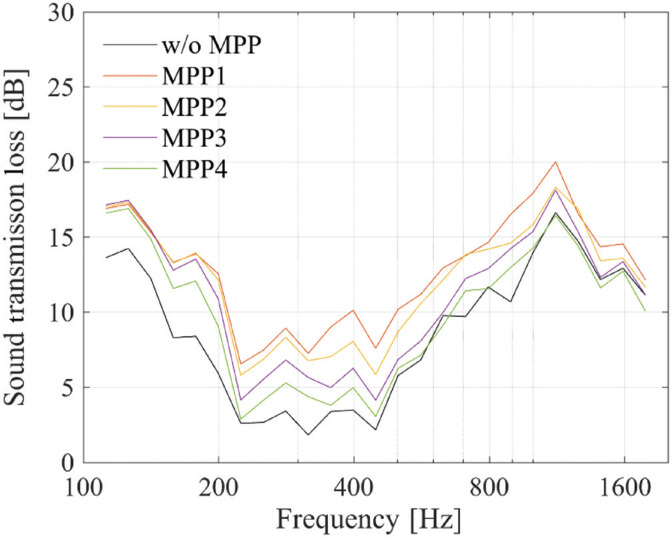
Sound transmission loss with MPP on the periphery of the cavity.

The results are shown in Fig. 6. Compared to the case without MPP (w/o MPP), the installed MPP1–4 generally led to an increase in *TL*. The amount of increase in *TL* was larger for cases with greater air back spaces, on which it is considered to be dependent on the sound absorption coefficient.

This trend is particularly pronounced around 300 Hz, where an increase of approximately 3–6 dB is observed. This indicates that the plenum door can be effectively designed to enhance *TL* in the target frequency range.

## Installation of MPP in the plenum door

In the previous section, the surfaces on the periphery of the cavity were treated as MPPs, with external air back spaces added to accommodate the installation of MPP (Fig. 5). However, in this section, the approach considers inserting MPP inside the plenum door, including the air back spaces.

### Installation to interior surfaces

**Figure 7:**
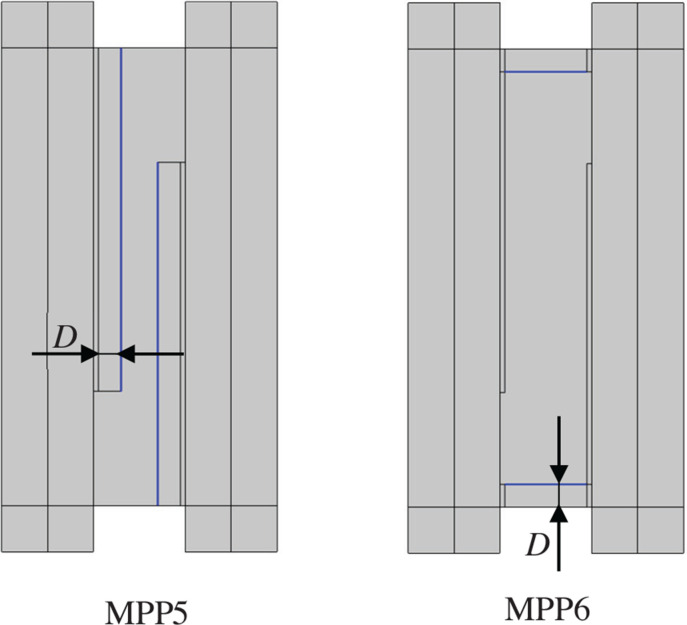
Model with MPP5 and MPP6 installed along the interior surfaces. *D* is the thickness of the air back spaces [mm].

An investigation was conducted on two configurations: one in which MPP was installed to the plenum door panels (MPP5) and another in which MPP was installed at the edges of the plenum door (MPP6). The cross-sectional diagrams for each configuration are shown in Fig. 7. The parameters of MPP were set to be the same as those of MPP4 in Table 2.

From the perspective of visibility and daylighting, visibility cannot be achieved in MPP5 because the MPP covers the panel surfaces; however, daylighting can still be obtained by utilising translucent or transparent materials for the MPP. In contrast, MPP6 is specifically designed to maintain visibility.

**Figure 8:**
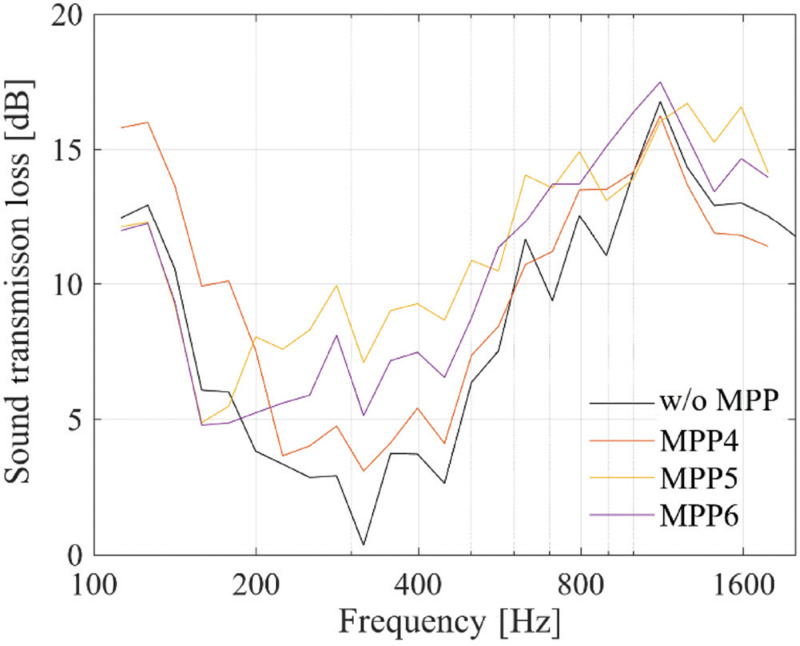
Sound transmission loss with MPP to interior surfaces.

The results are shown in Fig. 8. Compared to MPP4, which has the same thickness of the air back spaces, in the case of the MPP5 and MPP6 models, *TL* increases in the mid-to-low-frequency range. While the MPP4 model exhibits higher *TL* in the low-frequency range, around 300 Hz, the MPP5 and MPP6 models show higher values, suggesting improved sound insulation performance at the target frequency.

However, as MPP5 is installed along the panel, maintaining visibility may be challenging. Therefore, selection should be based on the intended application and the target frequency range.

### Installation in the middle of the cavity

**Figure 9:**
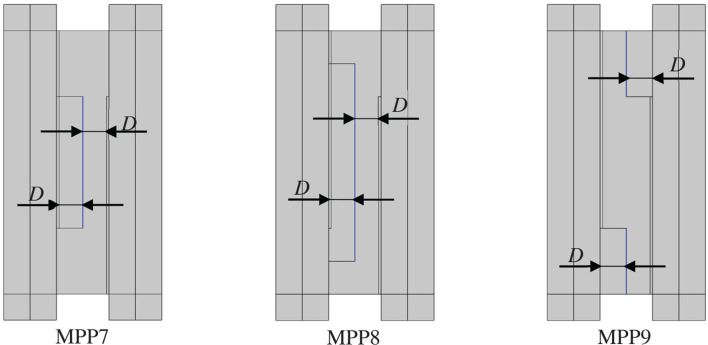
Model with MPP7, MPP8 and MPP9 installed at the centre. *D* is the thickness of the air back layer [mm].

This section discusses the case where MPPs are installed at the middle of the cavity, with the interior surfaces of the plenum door serving as the backing walls. The installation position of MPP was varied, and the cases were designated as MPP7 to MPP9 for analysis. The cross-sectional diagrams of each configuration are shown in Fig. 9, in which MPP is indicated in blue.

The parameters of the MPP used in this study were as follows: an air back space’s thickness of 90 mm, a hole diameter of 0.6 mm, a plate thickness of 0.6 mm and an open area ratio of 0.25%. The peak absorption frequency was set to approximately 300 Hz (Table 3).

**Table 3.** MPP parameter of MPP7-9

Air back space *D* [mm]	Hole diameter *d* [mm]	Panel thickness *t* [mm]	Porosity *p* [%]
90	0.6	0.6	0.25

**Figure 10:**
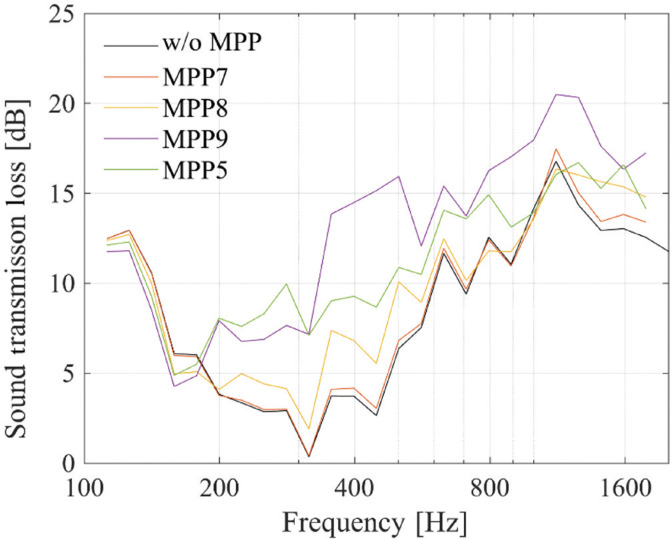
Sound transmission loss with MPP in the middle of the cavity. (The graph of MPP5 is included as a reference.)

The results are shown in Fig. 10. The graph of MPP5 is included as a reference. In the case of MPP7, *TL* did not increase significantly, whereas in MPP8 and MPP9, an increase was observed. Particularly in MPP9, the increase in *TL* was remarkable, indicating that MPP functions primarily near the opening.

**Figure 11:**
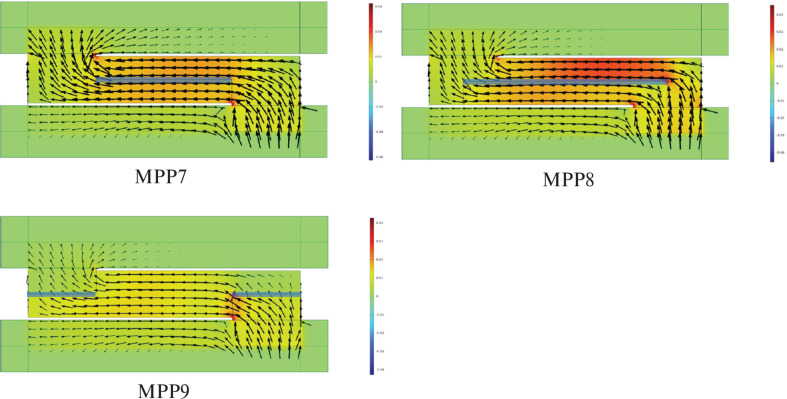
Acoustic intensity distribution for MPP7–8.

In order to clarify the positions where the MPP is most effective, the acoustic intensity distribution at 300 Hz is shown in Fig. 11. According to this figure, in MPP7 and MPP8, the sound waves flow parallel to the MPP surface, whereas in MPP9, a larger portion of the sound waves impinges on the MPP from a direction closer to normal incidence. This indicates that the sound field inside the plenum door exhibits directional propagation, and that the angle of incidence of the sound waves relative to the MPP surface influences the amount of sound absorption achieved by the MPP.

The amount of increase in MPP9 is higher than that in MPP5, suggesting that it is effective from both the perspectives of maintaining visibility and improving sound insulation performance.

## Concluding remarks

In this study, the effects of acoustic treatment and the installation of MPPs inside a plenum door on its sound insulation performance were examined. The results demonstrated that the treated area, absorption coefficient, air back space thickness and installation conditions all play significant roles in determining performance. However, several limitations of this study should be noted.

First, due to structural constraints, plenum doors tend to require a relatively large overall thickness to maintain sufficient strength and rigidity as functional doors. This may affect their practicality and architectural design, and it limits the degree to which acoustic treatment or MPP installation can be freely optimised. Moreover, the present study primarily focused on acoustic aspects: factors that can be dominant in real buildings – such as structural vibration and leakage paths through the frame – were not fully addressed.

Furthermore, although the results suggest that the effectiveness of MPPs depends not only on their area and the thickness of the air back space but also on their placement relative to the sound field inside the plenum, the optimal configuration could not be generalised. In particular, a more detailed analysis is required to clarify how the internal acoustic field of the plenum and the positioning of the MPPs interact to contribute to *TL*.

Overall, this study identified both the conditions under which acoustic treatment and MPPs are effective and those under which their effects are limited. At the same time, structural constraints and the complexity of the internal sound field present challenges for generalising an optimal design. Future work should further investigate structural aspects as well as the detailed characteristics of the internal acoustic field.

## References

[1] Torresin S, Albatici R, Aletta F, Babich F, Oberman T, Kang J (2019). Acoustic design criteria in naturally ventilated residential buildings: new research perspectives by applying the indoor soundscape approach. Appl Sci [online].

[2] Ford RD, Kerry G (1973). The sound insulation of partially open double glazing. Appl Acoust [online].

[3] Tang SK (2017). A review on natural ventilation-enabling façade noise control devices for congested high-rise cities. Appl Sci [online].

[4] Tong YG, Tang SK, Kang J, Fung A, Yeung MKL (2015). Full scale field study of sound transmission across plenum windows. Appl Acoust [online].

[5] Li X-L, Tang SK (2023).

[6] Tang SK, Leung M, Yip SSL, Lee RYC (2023).

[7] Maekawa Z, Rindel JH, Lord P (2011). Environmental and architectural acoustics.

[8] Fusaro G, Yu X, Lu Z, Cui F, Kang J (2021). A metawindow with optimised acoustic and ventilation performance. Appl Sci [online].

[9] Fusaro G, Yu X, Kang J, Cui F (2020). Development of metacage for noise control and natural ventilation in a window system. Appl Acoust [online].

[10] Sakagami K, Inoue H, Okuzono T (2023).

[11] Maa D-Y (1987). Micro-perforated-panel wideband absorbers. Noise Control Eng J [online].

